# Consumers' Perceptions of Patient-Accessible Electronic Medical Records

**DOI:** 10.2196/jmir.2507

**Published:** 2013-08-26

**Authors:** Christina Zarcadoolas, Wendy L Vaughon, Sara J Czaja, Joslyn Levy, Maxine L Rockoff

**Affiliations:** ^1^CUNY School of Public Health at Hunter CollegeNew York, NYUnited States; ^2^Department of Psychiatry and Behavioral SciencesUniversity of Miami Miller School of MedicineMiami, FLUnited States; ^3^Joslyn Levy & Associates, LLCNew York, NYUnited States; ^4^Department of Biomedical InformaticsColumbia UniversityNew York, NYUnited States

**Keywords:** electronic medical record, EMR, patient portal, usability, health literacy

## Abstract

**Background:**

Electronic health information (eHealth) tools for patients, including patient-accessible electronic medical records (patient portals), are proliferating in health care delivery systems nationally. However, there has been very limited study of the perceived utility and functionality of portals, as well as limited assessment of these systems by vulnerable (low education level, racial/ethnic minority) consumers.

**Objective:**

The objective of the study was to identify vulnerable consumers’ response to patient portals, their perceived utility and value, as well as their reactions to specific portal functions.

**Methods:**

This qualitative study used 4 focus groups with 28 low education level, English-speaking consumers in June and July 2010, in New York City.

**Results:**

Participants included 10 males and 18 females, ranging in age from 21-63 years; 19 non-Hispanic black, 7 Hispanic, 1 non-Hispanic White and 1 Other. None of the participants had higher than a high school level education, and 13 had less than a high school education. All participants had experience with computers and 26 used the Internet. Major themes were enhanced consumer engagement/patient empowerment, extending the doctor’s visit/enhancing communication with health care providers, literacy and health literacy factors, improved prevention and health maintenance, and privacy and security concerns. Consumers were also asked to comment on a number of key portal features. Consumers were most positive about features that increased convenience, such as making appointments and refilling prescriptions. Consumers raised concerns about a number of potential barriers to usage, such as complex language, complex visual layouts, and poor usability features.

**Conclusions:**

Most consumers were enthusiastic about patient portals and perceived that they had great utility and value. Study findings suggest that for patient portals to be effective for all consumers, portals must be designed to be easy to read, visually engaging, and have user-friendly navigation.

## Introduction

### Background

A number of social and economic factors, such as rising health care costs, a trend towards home health care, as well as shortages of health care workers, have encouraged consumers to increasingly assume a more active role in the management of their own health. Concurrently, advisory bodies, such as the Institute of Medicine, and government agencies have promoted the ability of health information technology to not only enhance the patient-centeredness of health care, but to improve the quality and efficiency of health care delivery overall [1].

One proliferating electronic health (eHealth) tool is the patient-accessible electronic medical record or patient portal. A patient portal, as defined by HealthIT.gov, is an Internet application that allows patients to access their electronic health records and communicate with their health care providers [2]. In this paper, we use patient portal to refer to a secure system tethered to a provider’s electronic medical record. The patient portal typically offers patients the ability to communicate with providers, manage medications, schedule appointments, review lab results, access medical history data, as well as provide links to obtain patient education or health information from other online sources [3]. Patient portals allow consumers to take greater control of their health information by changing traditional top-down (doctor to patient) methods of health communication [4] and improving satisfaction with provider communication and overall care [5]. While research is limited, some studies suggest benefits such as greater engagement in health care with online access to personal health information [6], improved rates of screening [7], improved appointment adherence [8], and greater sense of confidence and empowerment, increased knowledge about health, and improved health behaviors [9,10].

The Health Information Technology for Economic and Clinical Health (HITECH) Act has promoted the adoption of health information technology by incentivizing the meaningful use of electronic medical records. Tying incentive payments from Medicare and Medicaid to meeting meaningful use objectives, newly released requirements stipulate that patients must be given online access to their health record with the ability to access, print, share, or download their health information. In addition, providers will need to ensure that at least 5% of patients actively use this technology [11].

The study is motivated by no less than two factors. First, consumers are increasingly interested in accessing their personal health information online [12-16]. The Markle Survey on Health in a Networked Life reports that roughly 70% of the public and 65% of doctors believe that patients should be able to download and keep their own health information [17]. Walker and colleagues (2009) conducted focus groups with frequent Internet users and found that participants not only wanted electronic access to their medical records, but customized health information and advice as well [18].

Second, the literature has documented the vulnerability of certain population groups to disparities in health outcomes [19,20] and health care quality [21,22]. Vulnerable groups are most often described as racial and ethnic minorities, poor, under-educated, immigrants, and those lacking English proficiency [23]. Many vulnerable populations are also likely to be low literate and/or low health literate [24,25]. Strong evidence exists linking low literacy and low health literacy with poorer health behaviors, disease management skills, and health outcomes [26-31]. This is particularly concerning considering most health information created for the general public is written at or above the 10th grade level [32], with health websites often at an even higher level [33], even though more than half of the adults in the United States read at an 8th grade level and lower, and some vulnerable populations read at 5th grade level or lower [34].

Vulnerable populations continue to receive poor quality health care as well as face more barriers to receiving care than more advantaged groups, despite continuing efforts to reduce such disparities [22]. Some have suggested that use of eHealth, by providing increased access to health information and support, may help to ameliorate disparities in vulnerable or disadvantaged groups [23,35]. However, there is conflicting data regarding these consumers’ perceptions and the use of patient portals and other electronic health information.

Studies with medically underserved and vulnerable patients [36,37] found strong interest in accessing online health records, and research also shows that some vulnerable populations, such as racial and ethnic minorities, have similar or even greater interest in accessing health information online than national samples [38]. Other studies show that racial and ethnic minorities have lower rates of enrollment [39-41], logging on [42,43], likelihood of receiving an access code, and regularly using a patient portal [44] than non-minorities. However, once enrolled, studies found no disparities in use by race/ethnicity [39,45] or less of an association with race/ethnicity than with portal adoption [41]. These findings indicate that the way patient portals are designed and presented to consumers may influence how portals are perceived, valued, and ultimately utilized.

### Objective

Thus, our goal in this qualitative study was to identify what perceived utility and value vulnerable health consumers attach to the concept of patient portals and to core features and functions of these portals. We utilized focus groups targeting lower education level, minority residents of New York City to explore this question.

## Methods

### Participants

A total of 28 individuals, 10 male and 18 female, 21-63 years of age, with a high school education or less, participated in 4 focus groups held June-July 2010. Each of the 4 focus groups consisted of a convenience sample of 6-8 participants along with a moderator and a note taker. The groups were conducted using a semistructured format. Prior to commencing the focus groups, participants completed a technology experience questionnaire adapted from Czaja et al [46,47]. Each group lasted approximately 90 minutes. All focus groups were audiotaped. At the end of each group, participants were paid US $40.00 and given a US $4.50 round-trip NYC MetroCard for their time and travel. All study protocols and materials were approved by the Mount Sinai School of Medicine Institutional Review Board.

### Sample

Individuals were eligible to participate if they met the following criteria: New York City resident, aged 21-75 years, able to read and speak English, and no higher than a high school education/GED.

### Recruitment

Recruitment was conducted at 3 sites in New York City: Mount Sinai Medical Center, East Harlem; Queens Library, Long Island City, Queens; and CAMBA, Flatbush, Brooklyn. These organizations and locations were chosen for their access to diverse population groups in different urban neighborhoods in New York City.

Each site is located in a health service area designated as Medically Underserved and a Primary Care Health Professional Shortage area by the United States Department of Health and Human Services Health Resources and Services Administration (HRSA). According to HRSA, Medically Underserved Areas have “too few primary care providers, high infant mortality, high poverty and/or high elderly population” [48].

A description of the focus group neighborhood, site, and participants can be found in Table 1 [49-53].

We specifically recruited in Queens for 2 age groups: one 18-49 and the other, 50 and older. We chose this location because we believed they had the largest and most age-diverse pool of candidates meeting our criteria. Although there are a number of different age ranges within which researchers and others operationalize “younger” and “older” adults, we follow other studies and surveys, which have defined “older” adults as those 50 years and older [54-56].

Using IRB (Institutional Review Board) approved recruitment and advertising materials, staff members of these programs assisted with recruitment of participants by distributing flyers about the study, including the inclusion criteria, to their participants and patrons. Interested individuals either signed a sheet requesting researchers contact them to be screened for the study or called researchers directly. Potential participants were screened by research staff over the phone to ensure they were eligible for the study. Thirty-two individuals met eligibility criteria, agreed to participate, and were scheduled to attend a focus group. Twenty-eight participants actually attended a group. All participants provided informed consent according to IRB requirements.

### Procedure for Focus Groups

It is often the case in focus group research that participants are somewhat familiar with the topic of discussion. However, in this case, most participants were unfamiliar with the concept of patient portals and their specific capabilities. Therefore, the moderator provided a scripted introduction to patient portals including demonstrations using readily available informational videos as prompts. The videos were produced by a large health system, a managed care company, a multispecialty health care provider, and a federally qualified health center network—all of whom have been forerunners in the adoption of electronic medical records for patients. We selected clips from these organizations because they represented a broad range of possible entities from which patients may receive health care. The videos were created by these entities for their patients, so what participants saw was what a real world patient might encounter when being introduced to a portal for the first time.

The moderator’s guide focused on exploring the following four core functions of portals: scheduling appointments, managing medications, proxy functions, and reviewing lab results. These four functions were chosen because they are universally present in most patient portals. Participants were asked questions such as what they thought about the idea of portals in general and each of the four functions and how likely would they be to use the feature. Participants were also encouraged to ask questions and offer critiques of portal content and capabilities, as well as respond to a scenario that asked how a patient portal might be of use in keeping them healthy.

**Table 1:** Focus group sites and participants.

Neighborhood	Snapshot	Focus Group Site	Description	Participants
East Harlem, Manhattan	38% of East Harlem residents live below the poverty level and 69% have a high school education or less. The population is 55% Hispanic and 33% black/African American [49].	Mount Sinai Medical Center, Department of Health Education	The Department of Health Education provides comprehensive education and support programs in the community.	6 women
Long Island City, Queens	Long Island City is located in Northwest Queens. 20% of residents in this area live below the poverty level and 55% have a high school education or less. The population in Northwest Queens is 43% White, 28% Hispanic, 15% Asian, and 6% black/African American) [50]. The census tract in which the library is located is 44.9% Hispanic and 39.8% black/African American [51].	Queens Library, Long Island City Branch	Queens Library system serves the most ethnically diverse county in the United States and has the largest circulation of any US public library. The Long Island City branch provides a range of services, from adult learning and literacy programs to job readiness and computer training.	(1) 8 adults (2 men, 6 women), all between 18-49 years, and (2) 7 adults (2 men, 5 women), all 50 years and older
Flatbush, Brooklyn	21% of Flatbush residents live below the poverty level and 58% have a high school education or less. The population is 77% black/African American and 9% Hispanic [52]. Over half of Flatbush residents are foreign-born (51%), primarily (80%) from non-Hispanic Caribbean countries [52,53].	CAMBA	CAMBA is a community-based organization in Brooklyn committed to serving low-income, homeless, and immigrant populations, among other vulnerable groups.	7 adults (6 men and 1 woman)

### Analysis

All focus groups were written up in quick notes by both the moderator and observer independently within 24 hours. Quick notes record initial impressions as these can extinguish rapidly over time. The analysis team, consisting of the moderator and note taker, established a process for analysis that consisted of developing a coding guide as follows. We used a grounded theory (GT) model to analyze focus group data [57-59]. GT involves constantly comparing the data, coding, and identifying interchangeable indicators to reveal patterns that ultimately lead to categories. Two coders independently and repeatedly listened to the audio, referred to quick notes, and then conferred to discuss and refine emerging topics and themes. This process continued until the coding guide was finalized. The coding guide and accompanying narrative summary was developed to describe the key content of each group and enable a ready comparison of break characteristics, that is, the factors that differentiated one group from another.

All 28 participants had no higher than a high school degree, 15 had a high school diploma or GED, and 13 did not complete high school. Most were of ethnic/racial minority backgrounds, 7 Hispanic, 19 non-Hispanic black, 1 non-Hispanic white, and 1 Other, and all were of low economic means, including 8 with reported household incomes below US $20,000 (Table 2). Although more than half of participants refused or said that they did not know their annual household income, it is acknowledged that research participants may not provide income information because they are unsure of the answer or feel it is too private to share [60]. However, research has also shown that those who refuse to provide income information are more likely to not be working, have less education, and live in a low socioeconomic neighborhood than those who report income [61]. Given the neighborhood characteristics of our focus group locations, we are confident that our participants are representative of the vulnerable population we intended to reach.

**Table 2:** Participant demographics (N=28).

		East Harlem (n=6)	Brooklyn (n=7)	Queens (n=8)	Queens older adults (n=7)	Total (n=28)
**Age in years, mean (SD)**		26.7 (5.7)	37.1 (5.6)	38.0 (9.5)	56.6 (4.2)	40.0 (12.4)
**Gender, n**						
	Male	0	6	2	2	10
	Female	6	1	6	5	18
**Ethnicity/Race, n**						
	Hispanic	5	0	2	0	7
	Non-Hispanic white	0	0	0	1	1
	Non-Hispanic black	1	7	5	6	19
	Other	0	0	1	0	1
**Education, n**						
	Less than HS	3	2	5	3	13
	HS graduate/GED	3	5	3	4	15
**Income, n**						
	<$20,000	1	1	3	3	8
	$20,000+	3	0	1	1	5
	Don’t know / refuse	2	6	4	3	15
**Occupational status, n**						
	Work full-time	3	3	0	0	6
	Work part-time	1	0	0	0	1
	Student	0	0	3	0	3
	Homemaker	0	0	1	0	1
	Retired	0	0	0	3	3
	Seeking employment	2	4	4	2	12
	Other	0	0	0	2	2
**Primary language, n**						
	Yes (English)	6	0	8	7	21
	No (Other)	0	7	0	0	7
**General health, n**						
	Excellent	0	3	1	1	5
	Very good	1	0	2	1	4
	Good	3	3	3	3	12
	Fair	2	0	2	1	5
	Poor	0	1	0	1	2

## Results

### Technology in People’s Daily Lives

First, we describe participants’ current technology and eHealth use, record-keeping behaviors, and health information seeking behaviors. Then, we discuss participants’ responses to selected features of patient portals. Finally, we present themes arising from participants’ attitudes and perceptions of patient portals.

The majority of participants currently used communication technology such as computers, the Internet, and mobile devices. All reported that they were currently using or had used a computer in the past and only 2 participants reported having no Internet experience (Table 3). All participants had either a basic cell phone or smart phone (some participants used both) (Table 4).

Most participants used a computer at home, but many also accessed computers in a variety of other places, such as at a public library, adult learning center, friend or relative’s house, work, or community center (Table 5). Many used technology to perform tasks of everyday life such as sending email, accessing social media, playing games, and searching for information online (Table 6). Most participants were not only technology users but were interested in learning more ways to stay connected through technology.

The East Harlem group was the most facile and “on the grid” of all of our participants. All 6 women regularly reported using a computer and the Internet in their daily lives. Facebook, email, pictures, Myspace, shopping, Twitter, school websites to check their children’s progress, planning travel/trips, or finding their way around town were among the things that they routinely do online. The Queens older adults group reported that they were learning how to access new features on their mobile devices or computers, with several expressing a desire to learn how to “text” and others regularly using social media. Members of the Brooklyn group were somewhat less likely to have used some forms of technology (the only group with some members reporting no Internet experience—see Table 2). However, they were more anxious to learn about and embrace technology than the Queens older adults, perhaps because they saw mastery of technology as a key part of their acculturation and social and economic success.

Previous use of technology for health-related issues was limited across all groups. Only 10 of 26 Internet-using participants reported ever having searched for health information online (Table 6), in contrast to national statistics showing 80% of all online adults have used the Internet to search for health information [62]. However, there were a few participants who reported not only searching for health information, but accessing their own personal health information electronically. One participant (Queens 21-49) reported having access to a fully functioning patient portal, noting that she had a “health scare”, for which she regularly accessed a portal to help her “stay on top of my medical records”. Another participant in the Queens older adults group reported carrying his medical information on a jump drive attached to his keychain.

Most participants across groups recognized benefits in using technology and eHealth tools to manage their health care, although a few expressed concerns associated with these tools. Younger participants, such as those in the East Harlem group, were very comfortable with technology and saw the use of eHealth tools as an extension of what they were already doing in their everyday lives. They also expressed the most interest and excitement around features that would make their lives more “hassle free”, such as using the portal to make appointments and get copies of medical and immunization records for their children.

Likewise, participants accustomed to being online and using social media transferred their expectations for user-friendly formats and tools to the patient portals we showed them. Participants expressed a desire for features such as mouseovers/clicks for just-in-time information. They faulted a site for not having such common features. For some, having a website interface that resembled commonly used Internet sites affected how they felt toward the patient portals we were displaying. One participant summed this up by saying that if someone were trying to sell him on one of the systems we demonstrated, as an “average person”, he would pick the one that put “everything out front…it’s like working on Facebook or Hotmail, everything is in plain sight and I can deal with that and most people like that”.

**Table 3:** Use of computers and the Internet (N=28).

		East Harlem (n=6)	Brooklyn (n=7)	Queens (n=8)	Queens older Adults (n=7)	Total (n=28)
**Length of time using a computer, n**						
	> 5 years	6	2	6	4	18
	>1 year, but <5 years	0	1	0	1	3
	Between 6 months and 1 year	0	2	2	1	4
	<6 months	0	2	0	1	3
	No computer experience	0	0	0	0	0
**Experience with the Internet, n**						
	Internet experience	6	5	8	7	26
	No Internet experience	0	2	0	0	2

**Table 4:** Use of technology (N=28) of participants who use “frequently”.^a^

Technology	n (%)
Basic cell phone	26 (93)
Recording and playback device	20 (71)
ATM	17 (61)
TV set top box	16 (57)
Home telephone	13 (46)
Smart phone with Internet access	10 (36)
Computer/Video games	10 (36)
Digital photography^b^	10 (37)
MP3/iPod music player^b^	10 (37)
In-car navigation system	4 (14)
Automated movie ticket purchase kiosk	3 (11)
Fitness devices	3 (11)

^a^Percentages sum to more than 100% because participants could select multiple responses.

^b^One participant did not respond (n=27).

**Table 5:** Location of computer use (n=27^a^).^b^

Location	n (%)
Home	18 (67)
Public library	10 (37)
Adult learning center	9 (33)
Friend or relative’s house	9 (33)
Work	7 (26)
Community center	3 (11)
Other	3 (11)

^a^One participant did not respond.

^b^Percentages sum to more than 100% because participants could select multiple responses.

**Table 6:** Activities on the Internet % (n=26) of participants who use “frequently”.^a^

Activities on the Internet	n (%)
Email	17 (65)
Read the news online	13 (50)
Instant messaging^b^	12 (48)
Post resume or search for employment	12 (46)
Social media^b^	11 (44)
Find information about community events or religious services	10 (39)
Search for health information about an illness, or order medications or other health products online	10 (39)
Make reservations, search for maps, or get travel information online	10 (39)
Search for educational courses or materials, use instructional software, or participate in online degree training programs	9 (35)
Shop for clothes or other items, search for product information online	9 (35)
Download government forms or find information about benefits or programs^b^	8 (32)
Buy tickets, find information about shows, events or hobbies	5 (19)
Online banking and/or bill paying	4 (15)

^a^Percentages sum to more than 100% because participants could select multiple responses.

^b^One participant did not respond (n=25).

### Record-Keeping Behaviors

We asked people about what kind of record keeper they would say they were, in order to compare this information to their level of enthusiasm about an electronic medical record. No strong connection was found. The groups differed in self-description of their general record-keeping behavior. Most participants, with the exception of the East Harlem group, asserted that they kept records in their daily lives. Some participants, especially those in the Queens older adults group, noted that they kept “paper records”, for things like tax purposes and appointments. Several participants across groups noted that they kept records of their health information: “I keep medical records, my copies of labs and test results from doctors”. Another participant, as noted earlier, kept his medical information electronically on a jump drive.

### Sources of Health Information

When asked about sources of health information, participants reported using a mix of offline and online sources. Offline, participants reported going to the library, pharmacies, and health fairs, or, as one participant described “just talk[ing] to people who have a condition or a health care provider to get more information”. Of the online sources, WebMD and Google searches were most frequently mentioned as ways to obtain health information. However, although some participants commonly used the Internet to search for health information, several commented on the negative aspects of online health information. One participant described his experience with WebMD: “That thing is so hard to understand because they give you a schematic of a human body and you have to point to it but…it doesn’t really break it down where the average person can understand…some of the translation is in doctor terms, not in layman terms, so the average person that’s looking at it gets lost”. Others noted that Google searches result in “getting too much information” or finding searches “a little frustrating ’cause it’s not always exactly what you’re looking for, a specific answer to your problem”.

### Initial Participant Response to the Idea of Patient Portals

After being given a short overview of patient portals, participants were asked to respond to the concept of a portal by rating the importance of access to a portal on a scale of 1-5 (with 1-Not Important to 5-Very Important). Most people across groups rated access to a portal either Very Important or Important to them, with the exception of the Queens older adults group. Participants in the East Harlem group embraced the concept from the outset, with all members rating potential access to a portal as “5-Very Important”, identifying benefits in the use of technology and patient portals to manage health care for both their children and themselves. These women envisioned the benefit in having remote access to test results, immunization records for children, and reminders for upcoming appointments (“I think it is great”). The majority of participants in the Brooklyn and Queens (21-49) groups rated patient portals highly, either a 4 or a 5. Members of the Queens older adults group had the least initial enthusiasm towards portals. Only 1 participant in this group rated access to a patient portal as “5-Very Important”, with 4 participants rating access to patient portals as “1-Not Important”.

Next, the groups were shown short videos demonstrating appointment setting, health proxy functions, medication management, and lab test results from a selection of patient portals.

### Response to Patient Portals: “Housekeeping” Tasks

#### Appointments

Almost all participants were impressed with the convenience and control portals provided for making appointments with their health care provider. Many reported frustration with the interactive voice response (IVR) systems they encountered when calling their providers and appreciated the convenience and control offered by a portal—“This seems to give more control over when you would like to make an appointment”. However, some participants found the “drop-down menu” a barrier to use. They were not sure that the appropriate options would be listed in such a menu or that the available choices would allow them to fully describe the nature or urgency of their complaint—“I would just be concerned about if it’s verified enough what you could put, the reasoning, that’s the only thing…when you speak with [a staff person] they might help you be more specific”. Thus, although participants appreciated the utility of the appointment-making feature, they still wanted to know that they had the option of calling their provider’s office directly.

#### Health Proxy Function

Many participants saw value in having proxy access to the electronic records of their children or their parents. Functions that would enable them to monitor their relative’s health, manage the medications their relative was taking, and understand more about how a relative’s chronic conditions should be managed on a regular basis were seen as especially valuable. In reference to caring for her elderly parent, one participant commented, “I’m dealing with my father who’s been having a stroke. I’m second health proxy and there’s three of us…[it is] better for communication”. Mothers in the East Harlem group felt the proxy function would increase convenience, especially if the portal allowed them to access health and vaccination records on the spot. Said one mother, “I have two kids and just with school and everything, they need physicals, immunizations, everything, if I can access that online, it would just cut out so much of the back and forth [participant in background, ‘hassle’], to actually have to go to your doctor’s office and wait for an hour just to get their records”.

Some consumers in the Queens 21-49 and Queens older adults groups were more circumspect about the possibility of an adult child or other family member gaining access: “Suppose you, as a parent, don’t want your child to know what is going on with you?” Continued discussion, however, assuaged some of these concerns, for example, understanding that the proxy is a voluntary act and that patients did not have to allow anyone, including their grown children, electronic access to their record.

When participants pondered the issue of parental access to an adolescent’s online health records, participants across all groups, except for the Brooklyn group, found privacy and confidentiality issues complicated to resolve. One participant believed that a parent having access to their teenager’s patient portal could inhibit the teen from talking to his or her doctor: “It might just make them more hesitant to disclose things to their doctor if they know that their parents can view this… it might just make them not want to say things that they don’t want their parents to know”. Another participant noted that even in current medical practice, “with teenagers of a certain age…they do have something where the parents are not allow[ed] to see certain things”. Thus, many participants believed a portal’s proxy function could be useful but acknowledged that rules concerning appropriate access needed further consideration.

### Response to Patient Portals: Health Management Tasks

#### Medication Management

Use of a portal for medication management tasks, such as requesting medication refills, was not as popular a feature for participants. For some, the importance of the feature was not immediately apparent because, in the words of one participant, “I don’t get no medicine”. Many participants seemed more comfortable calling to get a prescription refilled or going directly to the pharmacy, noting that you may not always have access to a computer. However, the one participant who had access to a patient portal reported that she found this feature to be a great benefit, “With my doctor, if I need medication it goes straight to the pharmacy. It’s emailed right away. So it is excellent. Without having to wait for a paper prescription…By the time you get home you could pick your medicine up, so it’s very convenient”. Even those participants who did not feel that the medication management feature would be a benefit for them felt that it would be good for the “disabled and elderly”.

#### Lab and Test Results

**Figure 1:**
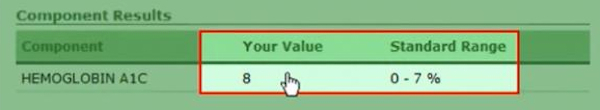
Sample hemoglobin test result from patient portal.

Access to lab and test results is a central function of most patient portals. Most participants said being able to see their test results was a very important feature for them. All groups viewed the following example of a lab test result from a patient portal (typical of test result formats we have reviewed) (Figure 1).

“Where is the standard?…The 0-7%, what exactly does that mean?” and “Is [that a] normal level for my blood?…I don’t know”. Participants wanted this page to be easier to use: “[be] more specific, like kind of in layman terms…Is that a good result? Is that a bad result?” or state clearly, “For a healthy person, it should be…you’re either above the window or below it”.

**Figure 2:**
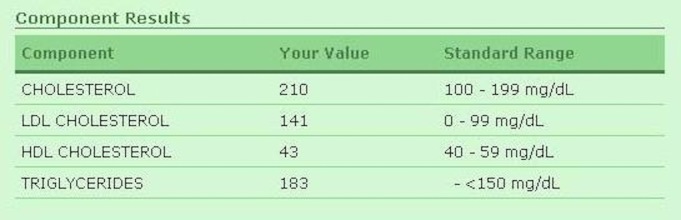
Sample cholesterol test result.

**Figure 3:**
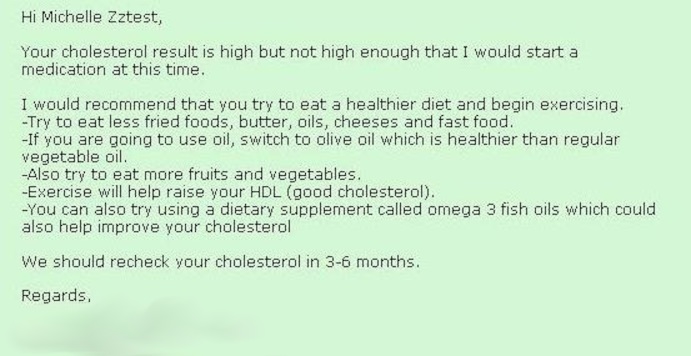
Provider's note accompanying cholesterol test result.

When participants were shown a test result that included a short explanatory note from the doctor (Figures 2 and 3), they felt they better understood the test result numbers and whether they should be concerned or not, noting that, “It’s written. It’s clear. You can look at it and understand it. Sometimes the doctor’s visit is so fast you might not even get as much as that [in your visit]”. Another said, “A lot of times…the doctor explain[s] things, but he don’t explain things…99% of the time when people go to the doctor they want to get in and out…with this here, I truly feel because they broke [it] down. Okay, this is high but…you have a reminder, you have something…that’s there that you could look at.”

For some consumers, however, the accompanying doctor’s explanation did not go far enough. They wanted the test results page to explain the doctor’s recommendations, for example, that the cholesterol test results were not high enough to require medication and to change their diet, and a further explanation of the different test components and the risks associated with certain values in a way they could understand—the why behind the recommendation. Looking at the sample results for a cholesterol test, one participant commented, “See this, the numbers…[referring to the doctor’s note] he just telling me that other part, making me feel comfortable, but I want to know what’s that 210 and…what’s the difference between the LDL and the HDL…break it down for me”.

### Key Themes

#### Consumer/Patient Empowerment—“Information is Power”

As reported above, most participants, with the exception of the Queens older adults group, were very interested in having access to their personal health information from the start. Many individuals expressed what can only be described as disbelief that the difficulties they historically have had accessing their medical records would be remedied. Participants recalled encountering cumbersome and daunting bureaucracies when they wanted a copy of their own medical records or that of a care recipient, such as a child or elderly parent (“Gotta go sign the paper, and you gotta wait a week, and then you gotta pick it up”). Others reported having to pay per page for copies of their medical record or were charged a co-pay to get test results.

#### Extending the Doctor’s Visit/Enhancing Communication With Health Care Providers

Participants almost unanimously named the short length of a doctor’s visit, the stress of not understanding things fully while at the visit, and the inability to access useful information post visit as characteristic of their health care experience. Patient portals were seen as offering at least a partial remedy for this very unsatisfactory situation. Many felt that online records could empower them with increased access to health information, resulting in a greater focus on their health and allowing them to be more proactive about taking care of their health.

The efficiency and portability of electronic medical records was seen as another huge benefit. Many participants said that their doctors used electronic medical records, and they felt that these records enhanced communication with providers: “When it was paper, when you go see a new doctor, they’re like asking you everything that you already given, all your demographics …when they can just look it up…and they’re not asking you all the same things all the time”. Although a few participants described themselves as “old school”, preferring in-person doctor-patient encounters without an electronic record (“the whole everything-on-the-computer thing is cool, it’s okay, but I’m kind of old school, I like the [personal]”) or accessing health records in paper format, there was also an acknowledgement that times change and with those changes would come greater acceptance of new technology (“For the next generation, it could be accepted…a lot of things that were thought about in the 60s is now being implemented in 2000”). Several participants, in fact, could foresee the benefits of enhanced communication with health care providers due to the portability of health information via patient portal: “I like it because if you go on vacation, you can get sick. And you have your medical record [and] you can give it to any doctor”. Another could see the utility of a portal to enable her to get more out of a doctor’s visit with her young children, “When I take my six-year-old to [an] appointment, I usually have my three-year-old there. They’re running around. You’re trying to listen to what the doctor’s telling you, but you’re still focused on, you know, your other kid. If you can go home and read everything…you’re more knowledgeable”.

#### Literacy and Health Literacy

Participants talked about the need to have patient portal information presented in ways “the average person can understand”. For example, after showing participants the sample lab test result page, one participant commented that she would use it “if I could read it and understand it…[but] I can’t understand it”. Another participant commented, “I just look and see numbers [referring to the sample results page]”. The reading and health literacy load (the demands on a reader’s understanding of science and other related concepts) of the sample lab test results in the demonstrated patient portal proved challenging and frustrating for participants. Participants found the reading and health literacy load of the content too high in 3 broad categories: (1) medical terms/medical information, eg, drug names, anatomy, chemistry, medical procedures (“There’s some people that don’t know the difference between good cholesterol and bad cholesterol. See, I didn’t know that until I got into my forties. Always just thought cholesterol. So that’s something they would have to explain”), (2) numeracy/numerical information, eg, number calculations, standard range, percentages (“Even if it does say…normal range, you’re still up by 1, your value, so that would kind of make some people freak out, like ok, what does that mean?”), and (3) design, navigation, and aiding tools, for which more than one participant suggested a design modification, such as an aiding tool that would allow you to click on the name of the test to get a more detailed explanation (“Could you click on each one and it can give you…a Wikipedia of it …?”).

#### Prevention and Health Maintenance

In the last segment of the focus groups, we asked participants to talk about what role portals might play in a user’s health. They commented that using the patient portal would allow people to “stay on top of [their] health”, “[focus on] prevention”, and “know more”. Although most were now positive about portals, they spoke in generalities about how a person’s health might improve as a result of using one. Most salient were the positive health impacts of reminders for appointments, annual visits, and screenings. Said one participant, “a lot of people don’t take charge of their health because they don’t even remember to take care of themselves, and a lot of times they don’t even know at what age they should be checking for what things”.

#### Privacy and Security Concerns

While privacy and security were not high priority concerns for most consumers we spoke with, in 3 of the 4 groups (with the exception of the Brooklyn group) there were a small number who voiced concerns related to the privacy and security of online records. An East Harlem participant, who asserted that access to a patient portal was very important, did so with the caveat: “If they are at your doctor’s office, you know anybody that has access to it, it’s like staff mainly, if it’s [a] website and…if there is some way someone can get your password…that’s a lot of information for someone else to have. Do you want to risk that?” A participant in the Queens 21-49 group feared that if someone was going through a divorce, an unhappy spouse might gain access and try to “damage the medical record” out of “spite”. Several members of the Queens older adults group feared “hacking” and identify theft; a couple of these participants initially felt that concerns over the privacy and security of electronic records would prohibit them from using a patient portal: “It comes down to a security point…the computer is awesome, I mean it’s off the chain. You could get on that computer, you could find out about anything, but do you really want your information up there?…I mean it’s a question”.

Some security and privacy concerns lessened once specific security features associated with patient portals were discussed, and participants identified the potential benefits of patient portals in their daily lives. Participants appeared willing to make tradeoffs, accepting the potential risk of breaches to their personal privacy for the convenience and accessibility of electronic records. Across groups, only one participant maintained throughout that privacy and security concerns would prevent her from using a patient portal, but she acknowledged portals should be an option for those who wanted them.

### Response to the Concept of Patient Portals With More Information: Post Demonstration

After participants had the opportunity to view and discuss all of the demonstrations, they were asked to revisit their initial opinions of patient portals. Most participants, who were either indifferent or negative about the value of portals at the outset, raised their opinion of the importance of access to a portal once they had an opportunity to view and discuss common features; this was especially true in the Queens older adults group. Others, awakened to the possibilities, looked to the possible future benefits: “Is it possible, after let’s say, like your relatives, a parent or what have you, pass away, are you able to have access so that doctors can see what diseases and things that run in the family or…predisposition to be exposed to certain things?” A few wanted to know when a patient portal would be set up by their health care providers, so they could begin to access their records electronically.

## Discussion

### Principal Findings

Online technologies continue to change society and our daily lives in many ways. We see this graphically in the exponential growth in consumer use of the Internet and mobile devices to access health information and services [63-66]. So too, the changes that patient electronic medical records portend are likely to change basic paradigms of patient provider interactions and the historic alignment of access to information.

Our goal in this study was to identify the perceived utility and value vulnerable health consumers attach to the concept of patient portals as well as to core features and functions of these portals: appointment setting, proxy functions, medication management, lab results, and preventive information. Our method was conducting a series of focus groups targeting lower education level, minority residents of New York City.

As expected, most consumers we spoke with were not familiar with a patient portal and only 1 participant out of 28 regularly used a portal. Despite this, most were positive about patient portals and positive perceptions increased over the course of the focus group discussion so that those less positive at the start, became more so as the groups evolved.

Our participants did not indicate that lack of Internet access or privacy when using public computers to access portals posed a potential barrier to portal use, as found elsewhere [36,67]. Nor did our participants appear to have issues resulting from a lack of computer skills or experience, as has also been reported [68]. This may be due the fact that all of our participants had a fair degree of prior experience with technology: all had previously used computers, almost all had experience using the Internet, and a majority had in-home Internet access.

As discussed in the Methods section, participants were introduced to a range of portal features and were asked to reflect on the utility and values of these features. Throughout the sessions, participants were engaged, asked frequent questions, and made extensive comments and critiques of specific features of portal features and functionality. Participants listened carefully to the descriptions and demonstrations of features, such as making appointments and reviewing lab tests. Then they posed their own questions, for example, “would you receive a confirmation of your appointment?”, “will the portal tell you whether that cholesterol number is ok?”, and, “if not, what you should do about it?” In contrast to perceptions by some consumers that it would be easy to learn how to use electronic medical records [36], participants in these groups identified a number of health literacy and usability barriers to patient portal use, such as complicated medical and numerical information, as well as a lack of aiding tools.

On a broader level, participants easily recognized the ability of electronic records to empower them through increased access to their own records and as a way to get more out of a doctor’s visit. Agarwal and colleagues found that patients who perceived electronic records as empowering had significantly higher intentions to use a portal [69]. In our study, only a few expressed concern that electronic medical records would diminish their relationship with their provider. Instead, most participants focused on the benefits of portal use, especially in light of their increasing frustration with the ever shortening time with the doctor and growing complexity of the health information presented in visits. Research suggests that even if patients were able to understand all of the information related to their visit, only about half of the information would be remembered [70,71], if remembered correctly at all [72]. Thus, introducing patient portals to these consumers allowed them to foresee using a portal as a tool to reduce the burden of remembering everything shared during a doctor’s visit by allowing access and retrieval of visit related information at their convenience. Changing perceptions so that more patients begin to view electronic medical records as an “extension” of the doctor’s visit and as a complementary tool, may ease concerns of those who worry that use of electronic records will supplant or depersonalize provider relationships.

The majority of participants judged their current methods of engaging with their health and “staying on top” of their health as in need of much improvement. Although participants offered up only a few tangible specifics, most perceived patient portals as a good tool for improving their knowledge and engagement in their health care and that of their loved ones.

### Limitations

As with all qualitative methods, focus groups are not representative of any larger group of people. It would be important and enlightening to conduct further qualitative study to see if these initial findings regarding interest, empowerment, most appealing functions, barriers to use, and other reactions hold up. Focus group participants did not readily introduce specific ways that their health behaviors might change as a result of using a patient portal. In part, this could be a product of the questions used in the moderator’s guide. We think it is likely that the artificial nature of the portal review in this setting did not provide enough specific and personalized content and context for participants to more substantively reflect on what they, as a real patient with their own health conditions, would most likely do with a patient portal.

### Conclusion

The intent and promise of patient portals is that they will help engage people with their health, improve preventive care behaviors, and permit better management of chronic conditions. A handful of recent studies have begun to examine patients’ uptake of portals as well as patients’ perceptions and assessments of their actual use. Consistent with the views of our study participants, users report finding great value in patient portals [73], a perception shared by a wide range of patients, including those with mental health and substance abuse issues [68] as well as patients with HIV [67]. In general, patients report that portals positively impact communication with providers, and improved knowledge, empowerment, and self-care [73,74].

However, despite the positive potential of portals suggested by these findings, there still remains relatively little exploration of vulnerable patients’ experiences with these systems. In order to realize these benefits for all consumers, it is important to better understand the perceived and real barriers and opportunities that vulnerable groups, so disproportionately impacted by health disparities, face in actively using patient portals. This project moves a step beyond surveys and analyses about consumer use of patient portals by employing exploratory focus groups to study the perceptions and opinions of vulnerable, low educational level, and ethnic minority consumers in underserved communities. The findings signal that there is little reason to assume vulnerable populations are not accessing patient portals because they do not see the value of having electronic access to their personal health information. To the contrary, once consumers were given the opportunity to view and experience portal functions, they became very interested and motivated. Most participants perceived patient portals as a way to finally obtain information that has, until now, been out of their reach; as they often said, “knowledge is power.” We hypothesize that this target population, in bringing the experience and perspective that is a manifestation of their historically marginalized status, is, perhaps, even more motivated to embrace this and other emergent technologies.

These focus group findings serve as one baseline representation of public opinion regarding patient portals. Further study of public perception and preferences surrounding patient portals can be used to better guide the introduction of this technology to patients and add argument to the importance of promoting patient portal adoption and use. For example, some have suggested that increasing mobile access may encourage initial adoption and uptake of portals, since minorities are more likely to use these devices for health applications [43]. In addition, our results demonstrate that portal content must be developed that accommodates the needs of lower education level, low literate, and low health literate users. Clearly, it is imperative that patient portals are designed and refined with the input of a diverse body of consumers in order to guard against continued barriers and poor access to information. If not, patient portals will remain an unused or underutilized tool for those who could potentially benefit the most.

## Abbreviations

CAMBA: a community-based organization in Brooklyn committed to serving low-income, homeless, and immigrant populations, among other vulnerable groups.
GT: grounded theory
HITECH: Health Information Technology for Economic and Clinical Health Act
HRSA: Health Resources and Services Administration
IRB: Institutional Review Board
